# A 50-Year-Old Man with Deteriorating Cognitive Function and Impaired Movement

**DOI:** 10.1371/journal.pmed.1000019

**Published:** 2009-01-27

**Authors:** Andrew J Larner

## Abstract

Andrew Larner discusses the diagnosis and management of a man referred to the Cognitive Function Clinic with a 12- to 18-month history of deteriorating memory.

## DESCRIPTION of CASE

A 50-year-old man was referred to the Cognitive Function Clinic with a 12- to 18-month history of deterioration in his memory. The symptoms reported by the patient and his wife included forgetting what he was supposed to be doing, occasional disorientation in place, misplacing objects, and word-finding difficulties, all of which had affected his occupational function as a high school teacher. His past medical history was unremarkable. In the family history, his mother was said to have had Alzheimer disease (AD) in her fifties and died in her sixties, but no further details were available to confirm or refute this. The patient's neurological examination was normal.

### Initial Investigations

On the Mini-Mental State Examination (MMSE; see Box 1 for brief descriptions of cognitive tests), the patient scored 26/30 with evidence of impaired delayed recall. Initial neuropsychological assessment showed no evidence of generalised intellectual loss, since the full scale IQ measured with the Wechsler Adult Intelligence Scale–Revised (WAIS-R) was 109, comparable to the predicted premorbid full scale IQ of 100 measured with the National Adult Reading Test (NART). He was within normal limits on tests of memory. In tests of executive function using verbal fluency and the Stroop test, the patient was within normal limits and showed no perseverative errors or rule breaks. Structural brain imaging with computed tomography (he could not tolerate magnetic resonance brain imaging) was within normal limits. Despite these apparently reassuring results, follow-up for longitudinal assessment was recommended, in part because the symptoms affected occupational function and in part because of the family history.

**Box 1: Description of Cognitive Tests**

**Mini-Mental State Examination (MMSE)**

A brief (5–10 minutes) mental status questionnaire assessing attention, orientation, memory, language, and visuospatial copying.

**Wechsler Adult Intelligence Scale–Revised (WAIS-R)**

Widely used test battery of general cognitive abilities, deriving a full scale IQ from six subtests that comprise Verbal IQ and five subtests that comprise Performance IQ. Administration time 1–2 hours; may require more than one consultation if patients become fatigued.

**National Adult Reading Test (NART)**

A reading test of 50 irregularly spelled words. Since this function is relatively preserved in some forms of dementia, the NART may be used to estimate premorbid cognitive function, since longitudinal assessments of IQ are almost never available in initial clinical evaluation.

**Verbal Fluency Tests**

Generation of words beginning with a particular letter (Letter or Phonemic fluency), or exemplars of a particular category (Category or Semantic fluency) in one minute; tests of executive function.

**Stroop Test (Colour–Word Interference Test)**

Reading names of colours printed in inks of different colour; reading the names is much easier than reading the conflicting colours (inhibition task); test of executive function.

**Addenbrooke's Cognitive Examination**

A development of the MMSE addressing some of its shortcomings, with more detailed tests of memory, language, visuospatial, and executive function, and hence longer to administer (15–20 minutes).

**Graded Naming Test**

Patient must name 30 black-and-white line drawings, sequenced in order of difficulty; a test screening for naming deficits.

Over the next two years, the patient and his family noted further decline in memory function. New symptoms included repetitive questioning and difficulty remembering tasks to be done. He had to give up work, and his wife took over running the household finances. At reassessment, MMSE score was stable, but on the Addenbrooke's Cognitive Examination he scored 81/100 with evidence of impaired delayed recall. Repeat neuropsychological assessment showed a decline in intellectual performance (37 point fall in WAIS-R Verbal IQ) and deterioration in all measures of auditory and visual memory for immediate and delayed recall, but with normal working memory. There was also deterioration in word-finding and naming abilities (Graded Naming Test) and in tests of executive function (verbal fluency, Stroop test). Computed tomography brain imaging was now reported to show atrophy of the temporal lobes.

### What Is the Differential Diagnosis?

After two years of follow-up and investigation, this man fulfilled clinical diagnostic criteria for the diagnosis of dementia as enshrined in the *Diagnostic and Statistical Manual of Mental Disorders* [1], namely “the development of multiple cognitive deficits that include memory impairment… sufficiently severe to cause impairment in occupational or social functioning”.

The differential diagnosis of the cause of dementia is potentially broad (Box 2), although the majority of cases result from AD, with or without concurrent cerebrovascular disease. In dementia of young onset, arbitrarily defined as clinical onset before 65 years of age, the differential diagnosis is more varied [2,3]. Syndromes of frontotemporal lobar degeneration (FTLD) are more common, and indeed may be equal in frequency to AD in this age group [4]. In FTLD there is focal brain degeneration, which may show a predilection for the frontal lobes presenting with a behavioural phenotype, the behavioural or frontal variant of frontotemporal dementia, or for the temporal lobes presenting with a linguistic phenotype, either progressive non-fluent aphasia (PNFA) or semantic dementia. Overlap between FTLD and motor neurone disease, and movement disorders such as corticobasal degeneration and progressive supranuclear palsy, is also recognised [5]. Memory complaints are not uncommon in patients with FTLD, and many patients meet current clinical diagnostic criteria for AD [6]. However, memory deficits may be a relatively minor feature in FTLD, and standard screening tests such as the MMSE are insensitive to the diagnosis. Clinical suspicion should be raised when the extent of functional impairment appears inconsistent with the degree of cognitive deficits, requiring evaluation for cognitive deficits other than memory. Psychiatric illness, particularly depression, also enters the differential diagnosis.

**Box 2: Differential Diagnosis of Dementia**

- Neurodegenerative disorders:
  ○AD +/− cerebrovascular disease
  ○Parkinson disease dementia/dementia with Lewy bodies
  ○FTLDs:
    Behavioural variant frontotemporal dementia
    PNFA
    Semantic dementia
  ○Prion diseases
- Vascular dementias
- Alcohol-related dementia/Wernicke-Korsakoff syndrome
- Structural brain disease:
  ○Severe traumatic brain injury
  ○Tumours +/− radiotherapy
- Inflammatory brain disorders, e.g., multiple sclerosis
- So-called “reversible causes” (very rare):
  ○Neurosyphilis
  ○Hypothyroidism
  ○Vitamin B12 deficiency

Other conditions that need to be considered include vascular dementias, alcohol-related dementia, and traumatic brain injury. There are many neurological disorders that may also be attended with cognitive impairment, sometimes of sufficient severity to mandate a diagnosis of dementia. These so-called secondary dementias often present with other neurological signs in addition to dementia, such as parkinsonism, myoclonus, seizures, chorea, ataxia, and sensory signs.

Dementias inherited as monogenic Mendelian disorders (Box 3) are also more common with young-onset disease, although still rare. Examples include AD associated with mutations in the presenilin 1, presenilin 2, or amyloid precursor protein genes, FTLD associated with mutations in the tau or progranulin genes, and Huntington disease [7].

**Box 3: Differential Diagnosis of Dementia Syndromes Inherited as Monogenic Mendelian Disorders**

- Inherited dementias:
  ○Huntington disease
  ○Familial AD
  ○Familial FTLD
  ○Inherited prion disease
- Inherited vasculopathies:
  ○Cerebral autosomal dominant arteriopathy with subcortical infarcts and leukoencephalopathy
  ○Familial British dementia, familial Danish dementia
- Inherited movement disorders, ataxias, metabolic disorders that may be complicated by cognitive decline and dementia:
  ○Wilson disease
  ○Dentatorubropallidoluysian atrophy
  ○Presenile dementia with bone cysts
  ○Some spinocerebellar ataxias
  ○Some hereditary spastic paraplegias
  ○Neurodegeneration with brain iron accumulation

### Initial Formulation and Treatment

A provisional diagnosis of probable young-onset familial AD was made, based on the NINCDS-ADRDA (National Institute of Neurological and Communicative Diseases and Stroke–Alzheimer's Disease and Related Disorders Association) clinical diagnostic criteria of AD [8], and the family history of one other affected individual. The stringent definition of autosomal dominant disease transmission requires a family history with at least three affected individuals in at least two generations [9], which was not known to be the case in this family.

The patient was treated with cholinesterase inhibitors, licensed for the treatment of mild-to-moderate AD in this jurisdiction, but these produced no subjective or objective benefit despite switching between the different cholinesterase inhibitors [10]. Two years after diagnosis the patient completed independent screening for a pharmaceutical company–sponsored trial of a potential new AD medication by meeting all specified inclusion and exclusion criteria.

### Clinical Progress

At the age of 56, about seven years after the first onset of symptoms and four years after the diagnosis of probable AD, the patient developed new neurological symptoms and signs. He complained of a stiff neck, falls both forward and backward, occasional choking, and difficulty articulating (as opposed to finding) his words. Increasing apathy was also noted retrospectively, a possible and easily overlooked cause of his initial occupational difficulties. Neurological examination was now abnormal, showing slowed horizontal saccadic eye movements with reduced amplitude vertical saccades, dysarthria, a broad-based gait with postural instability, and a positive applause sign.

### Further Formulation and Investigations

Had it not been for the presence of dementia of Alzheimer type, these new non-cognitive neurological symptoms and signs were judged to be consistent with a diagnosis of probable progressive supranuclear palsy (PSP), based on NINDS-SPSP (National Institute of Neurological Disorders and Stroke–Society for Progressive Supranuclear Palsy) diagnostic criteria [11]. The chance concurrence of two diagnoses, probable AD and PSP, was thought unlikely and hence, seeking a more parsimonious explanation, neurogenetic testing of the tau gene was undertaken. This testing showed the patient to be heterozygous for the splice site mutation C915 +16 C > T at the exon 10/intron 10 boundary (denoted 10 +16), indicating a diagnosis of frontotemporal dementia with parkinsonism linked to chromosome 17 (FTDP-17) (Online Mendelian Inheritance in Man, catalogue entry +157140) [12,13].

Shortly thereafter, the patient developed a chest infection and died. Permission for autopsy was not granted, but the patient's family has given written informed consent (as outlined in the PLoS consent form) for the publication of the details of his case.

## DISCUSSION

Although PSP and AD may rarely occur together, in this case the clinical features and confirmatory investigations eventually led instead to a single diagnosis of FTDP-17 with a tau gene mutation. AD and PSP are usually distinct conditions. Although both are neurodegenerative disorders that may cause dementia, typically the pattern of cognitive deficits differs: in AD there is prominent amnesia, with impaired encoding of new information, with or without agnosia, aphasia, and apraxia; whereas in PSP there is slowness of thought, altered personality with apathy, and forgetfulness with impaired retrieval of information [14].

Prior reports of the signs of PSP developing in AD have not been identified; eye movement abnormalities, nuchal rigidity, and early falls are not features of AD, although parkinsonism may occur, typically in the later stages. There have been occasional reports of AD pathology in PSP and of AD/PSP overlap cases [15–17]. PSP has previously been reported to “evolve” on occasion in patients initially diagnosed with the PNFA phenotype of FTLD [18,19], but this patient did not have a PNFA phenotype.

Mutations in the gene encoding microtubule-associated protein tau (MAPT) typically present with a frontotemporal dementia phenotype with or without a parkinsonian syndrome—hence the clinical nomenclature of FTDP-17 [12]. Phenotypic heterogeneity has been reported in association with *MAPT* mutations: for example, an AD-like phenotype has been reported on occasion [20], including cases with the splice site 10 + 16 mutation [21,22]. This particular mutation has also been identified in a patient presenting with apparently sporadic young-onset PSP [23]. The current case suggests that tau gene analysis should be undertaken in individuals with a phenotype initially suggestive of AD who develop signs suggestive of PSP, as well as in patients with a diagnosis of “young-onset familial AD” who prove negative for mutations in genes deterministic for AD (amyloid precursor protein, presenilin 1 and 2) [22]. No specific treatments for frontotemporal dementia and progressive supranuclear palsy are available, but symptomatic drug and non-pharmacological treatments are sometimes tried.

**Key Learning Points**

- Longitudinal patient assessment may be required to determine the true significance of subjective memory complaints.
- The differential diagnosis of young-onset dementias differs from that of late-onset disease, with both FTLD syndromes and inherited dementias being more common.
- Emergence of neurological signs in young-onset dementia raises suspicion of a non-Alzheimer or inherited dementia syndrome.
- Concurrence of movement disorder characterised by parkinsonism and young-onset dementia should suggest the possibility of a mutation in the gene encoding MAPT.

With the benefit of hindsight, the initial misdiagnosis in this patient might possibly have been avoided by more detailed initial neuropsychological assessment, and by use of other neuroimaging modalities, both structural and functional. Although temporal lobe atrophy can be seen in both AD and FTLD, the distribution of focal atrophy can be diagnostically helpful, and functional imaging may show different distributions of hypoperfusion (single-photon emission computerized tomography or SPECT scanning) and hypometabolism (positron emission tomography or PET scanning) [5].

## Abbreviations

AD: Alzheimer disease
FTDP-17: frontotemporal dementia with parkinsonism linked to chromosome 17
FTLD: frontotemporal lobar degeneration
MAPT: microtubule-associated protein tau
MMSE: Mini-Mental State Examination
NART: National Adult Reading Test
PNFA: progressive non-fluent aphasia
PSP: progressive supranuclear palsy
WAIS-R: Wechsler Adult Intelligence Scale–Revised
